# *In vitro *tooth whitening effect of two medicated chewing gums compared to a whitening gum and saliva

**DOI:** 10.1186/1472-6831-8-23

**Published:** 2008-08-11

**Authors:** Michael Moore, Nathalie Hasler-Nguyen, Geoffrey Saroea

**Affiliations:** 1 Health Science Research Center, Indiana University – Purdue University Fort Wayne, 2101 Coliseum Blvd East Fort Wayne, USA; 2 Department of Preclinical Development, Novartis Consumer Health, Rte Etraz, Nyon, Switzerland; 3 Department of Medical Affairs, Novartis Consumer Health, Argentia Road, Mississauga L5N 2X7, Canada

## Abstract

**Background:**

Extrinsic staining of teeth may result from the deposition of a variety of pigments into or onto the tooth surface, which originate mainly from diet or from tobacco use. More recently, clinical studies have demonstrated the efficacy of some chewing gums in removing extrinsic tooth staining. The aim of this study was to assess the effectiveness of two nicotine medicated chewing gums (A and B) on stain removal in an *in vitro *experiment, when compared with a confectionary whitening chewing gum (C) and human saliva (D).

**Methods:**

Bovine incisors were stained by alternating air exposure and immersion in a broth containing natural pigments such as coffee, tea and oral microorganisms for 10 days. Stained enamel samples were exposed to saliva alone or to the test chewing gums under conditions simulating human mastication. The coloration change of the enamel samples was measured using a spectrophotometer. Measurements were obtained for each specimen (average of three absorbances) using the L*a*b scale: lightness (L*), red-green (a) and yellow-blue (b).

**Results:**

Medicated chewing gums (A and B) removed a greater amount of visible extrinsic stain, while the confectionary chewing gum with a whitening claim (C) had a milder whitening effect as evaluated by quantitative and qualitative assessment.

**Conclusion:**

The tested Nicotine Replacement Therapy (NRT) chewing gums were more effective in the removal of the extrinsic tooth stain. This visible improvement in tooth whitening appearance could strengthen the smokers' motivation to quit smoking.

## Background

An attractive smile and healthy looking teeth reflect well being and quality of life for the majority of people [1]. The natural color of a permanent tooth is determined by the enamel translucency enabling the underlying dentine color to be visible. Enamel acts as a filter for dentine by the way light passes through to the dentine and as the light is reflected back by the dentine, which is the tooth color visually perceived [2]. This color can be quantified with a spectrophotometer using the L*a*b* color scale, which measures lightness for L*, red-green color range for a* and yellow-blue range for b*. Coloration of teeth is due to extrinsic stains, which lie on the surface of the tooth and within the acquired pellicle, while intrinsic stains lie within the dental tissues [3]. The aesthetic effects created by both types of discoloration are issues, which are dealt with by the dentist. Most intrinsic stain cannot readily be removed, but can be masked by restorative techniques, or by chemical means such as peroxide. On the opposite, extrinsic staining can often be removed by simple mechanical actions [3]. Home care procedures that remove extrinsic stain, are focused on dentifrice and toothbrushes. More recently, some chewing gums with a tooth whitening claim have been launched with clinical studies supporting their efficacy in removing extrinsic tooth stains [4].

Chewing gum has also been used to deliver therapeutic agents such as nicotine for smoking cessation therapy [5]. The aim of this *in vitro *method described by Kleber [6] was to evaluate the extrinsic stain removal capabilities of two medicated chewing-gums containing nicotine, one confectionary chewing-gum with a tooth whitening claim and formulation, against a non chewing-gum negative control using only human saliva.

## Methods

### Products

The experimental chewing gums were provided by the sponsor in coded packaging, which is equivalent to blind test samples.

The tested products were two nicotine replacement therapy (NRT) brands, Nicotinell Mint coated chewing gum with 2 mg nicotine (chewing gum A; Novartis Consumer Health) and Nicotinell Thrive Mint chewing gum with 2 mg nicotine (chewing gum B; Novartis Consumer Health). The differences between the two gums are that chewing gum A has a 60% higher calcium carbonate content and a different Mint flavor base than chewing B. A confectionary chewing gum with a tooth whitening claim, V6 White Strong Mint (chewing gum C; Fertin), containing carbamide and xylitol but not polyphosphates was used as positive control.

### Preparation of enamel specimen

Four mm squares of dental enamel from bovine permanent incisors, which are a representative model for human teeth [7], were cut using a diamond cutting disk. Using a mold, an enamel square measuring 4 mm × 4 mm, was embedded in clear polyester casting resin to provide a 1.5 cm square block with the tooth enamel labial surface exposed. The top surface of the polyester block was flattened to the level of labial surface of the enamel square using a dental model trimmer, while using water as a lubricant. The surface was then smoothed by hand-sanding, on 400 grit emery paper using water as the lubricant until all grinding marks were removed. Finally, the top surface of the block was hand-polished to a mirror finish using a water slurry of GK1072 calcined kaolin (median particle size = 1.2 microns) on a cotton cloth. The finished specimen was examined under a dissecting microscope and discarded if surface imperfections were observed.

In order to render the polished tooth surfaces more similar to natural teeth and promote the formation of stain on the enamel, the specimens were etched for 60 seconds in 0.2 M HCl followed by a final etch with 1% phytic acid for 60 seconds. The specimens were then rinsed with deionized water and attached to the staining apparatus.

### Tooth staining apparatus

**Figure 1:**
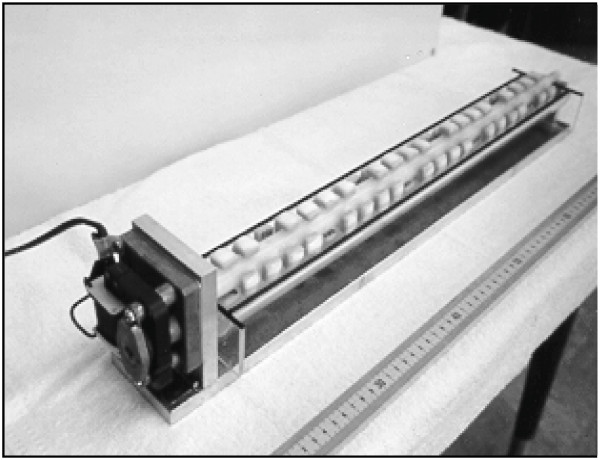
**Tooth staining apparatus**. The apparatus consists of an aluminum platform base, which supports a Teflon rod (3/4 inch in diameter) connected to an electric motor, which by means of a speed reduction box, rotates the rod at a constant rate of 1.5 rpm. Threaded screw holes are spaced at regular intervals along the length of the rod. The enamel specimens are attached to the rod by first gluing the head of a plastic screw to the back of the specimen, then screwing the enamel sample onto the rod. Beneath the rod is a removable, 300 ml capacity trough which held the tooth staining broth.

The tooth staining apparatus as shown in Fig. 1 was designed to provide alternate immersion into the staining broth and air-drying of the specimens. This instrument was made *in house*.

### Tooth staining broth preparation

The staining broth was prepared by adding 1.02 g of instant coffee, 1.02 g of instant tea, and 0.75 g of gastric mucin to 250 ml of sterilized trypticase soy broth [6]. Approximately 50 ml of a 24-hour *Micrococcus luteus *culture, which is a stain inducing bacteria found in the oral cavity was also added to the stain broth to promote and accelerate the extrinsic stain formation on the enamel specimens. The apparatus, containing the attached enamel specimens and staining broth, was then placed in an incubator at 37°C with the specimens rotating continuously through the staining broth and air. The staining broth was replaced with fresh broth once every 24 hours for ten consecutive days. With each broth change, the trough and specimens were rinsed and toothbrushed with deionized water to remove any loose deposits. On the eleventh day the staining broth was modified with the addition of 0.03 g of FeCl_3_.6H_2_O, and this was continued with daily broth changes until the stain on the specimens was sufficiently dark (L* < 35). The specimens were then removed from the staining broth, toothbrushed thoroughly with deionized water, and refrigerated in a humidor until used.

### Stain measurement

The color of the extrinsic stain on the enamel sample was measured by taking diffuse reflectance absorbance readings with a Minolta CM-503i Spectrophotometer with diffuse illumination/8° viewing angle, 3 mm aperture and with a D65 illuminant setting (Minolta Camera Co., 101 Williams Drive, Ramsey, NJ, 07446).

Absorbance measurements over the entire visible color spectrum were obtained using the CIELAB color scale [8]. This scale quantifies color according to 3 parameters, L* (lightness-darkness scale), a* (red-green chroma), and b* (yellow-blue chroma). In order to obtain reproducible readings, the stained enamel specimens were allowed to air-dry at room temperature for 30 minutes before measurements were made. These measurements were conducted by aligning the center of the 4 mm squared segment of stained enamel, directly over the 3 mm diameter targeting aperture of the spectrophotometer. An average of 3 absorbance readings using the L*a*b* scale were taken for each specimen.

### Test procedure

**Figure 2:**
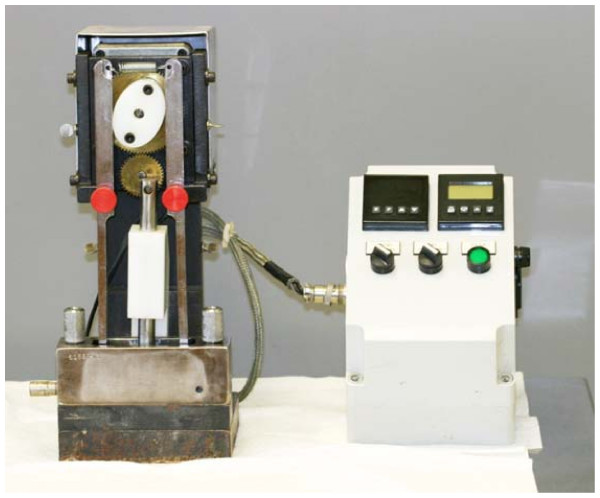
Mastication device.

Before the treatment, the baseline L*a*b* stain scores of the enamel specimens were determined and used to distribute the enamel samples into 4 balanced groups of 16 specimens each. A mastication device (Gildea Tool & Engineering Co., Inc. Fort Wayne, IN., USA), developed by Kleber *et al.*[6] to simulate the human mastication of chewing gum, was used (Fig 2). The stained enamel samples were immersed in 15 mL of freshly, stimulated human saliva (arising from *Parafilm*^® ^chewing), which had been collected and placed in the mastication reservoir. The thermostatically-controlled heating element was turned on to maintain the chewing gum and saliva at body temperature for proper chewing consistency. Fresh chewing gum (2.5 grams) and saliva were used for each 20 minute treatment period. Following the 3^rd ^(60 min) and 6^th ^treatments (120 min), the specimens were rinsed, allowed to dry for 30 minutes before a diffuse reflectance absorbance measurement was taken. After the final stain measurements, the specimens were pumiced using a dental handpiece in order to clean all residual stain from the enamel sample, and readings were taken again to obtain each specimen's inherent value for the "percent removed" calculation.

### Stain calculations

The overall change in the color of the stained enamel sample was calculated using the CIELAB equation: ΔE = [(ΔL*)^2 ^+ (Δa*)^2 ^+ (Δb*)^2^]^1/2^.

The ΔE value summarizes the overall change for each color factor ΔL*, Δa*, and Δb*) while ΔL and represents only the value for lightness. Both depict the ability of the test chewing gum to remove stain The data were calculated and defined as follows:

Stain Removed = ΔE or ΔL score after treatment.

Total Stain Available = ΔE score after treatment and pumicing.

% Stain Removed = "Stain Removed" divided by "Total Stain Available."

Each component of the L*a*b* scale represent the specific changes in the lightness (L*), red-green color (a*), and yellow-blue color (b*).

### Statistical Analysis

The difference in mean values calculated for the stain removal effect was determined and were statistically tested by analysis of variance for testing of differences between the groups using the Student Newman-Keuls test. All comparisons were tested at an overall of 0.01 significance level using the two sided t-test.

## Results

### Stain removal

Bovine tooth, a representative model for human teeth [7], was exposed to freshly stimulated human saliva with or without chewing gum products for 3 (60 min) to 6 cycles (120 min) of 20 min each. The confectionary whitening gum and saliva were renewed after every 20 minutes in order to simulate the average time that gum is normally chewed. Under these conditions, the extrinsic stain removed, which is evaluated as the overall color change (ΔE) between baseline and 60 or 120 minutes is shown in Table 1.

**Table 1:** Changes in total color (ΔE) of extrinsic dental stain

ΔE	A Nicotine chewing gum	B Nicotine chewing gum	C Whitening chewing gum	Saliva
60 min	10.80 ± 1.96**	10.08 ± 1.69**	5.00 ± 2.27*	1.03 ± 0.43
120 min	14.91 ± 2.12**	13.15 ± 1.57**	6.55 ± 2.24*	0.65 ± 0.29

Total color change (ΔE) in extrinsic stain scores by chewing gums A, B and C and saliva after 60 min or 120 min of *in vitro *mastication (mean ± standard deviation of 16 samples (n = 16)). Values with the same number of asterisks are not statistically different. Gums A and B versus C or saliva, as well as C versus saliva, are significantly different (at p < 0.01) at both 60 and 120 min.

The statistical analysis demonstrated that, after 60 minutes of mastication, chewing gum A resulted in a ΔE score of 10.80 ± 1.96 and chewing gum B resulted in a ΔE score of 10.08 ± 1.69. Both of them removed significantly (p < 0.01) more stain than chewing gum C (ΔE = 5.00 ± 2.27) and the saliva control (ΔE = 1.03 ± 0.43). In addition, chewing gum C had a significantly (p < 0.01) higher stain removal effect than saliva. After 120 minutes of mastication, chewing gums A and B continued to remove significantly (p < 0.01) more stain (ΔE = 14.91 ± 2.12 and 13.15 ± 1.57) than both chewing gum C (ΔE = 6.55 ± 2.24) and the saliva control (ΔE = 0.65 ± 0.29). Chewing gum C, once again, had a significantly (p < 0.01) higher stain removal effect than saliva.

To define the color of enamel calculated from the reflectance values, L* measures lightness on a scale from 0 (black) to 100 (snow white), thus contributing mostly to the whitening appearance of the tooth. ΔL values are demonstrated in Table 2 and correlated well with the ΔE observed in Table 1.

**Table 2:** Changes in lightness (ΔL) of extrinsic dental stain

ΔL	A Nicotine chewing gum	B Nicotine chewing gum	C Whitening chewing gum	Saliva
60 min	9.53 ± 1.77**	8.74 ± 1.73**	3.99 ± 2.13*	-0.16 ± 0.55
120 min	13.76 ± 2.32**	11.97 ± .1.65**	5.52 ± 2.26*	0.04 ± 0.48

Lightness change (ΔL) in extrinsic stain scores by chewing gums A, B and C and saliva after 60 min or 120 min of *in vitro *mastication (mean ± standard deviation of 16 samples (n = 16)). Values with the same number of asterisks are not statistically different. Gums A and B versus C or saliva, as well as C versus saliva, are significantly different (at p < 0.01) at both 60 and 120 min.

**Figure 3:**
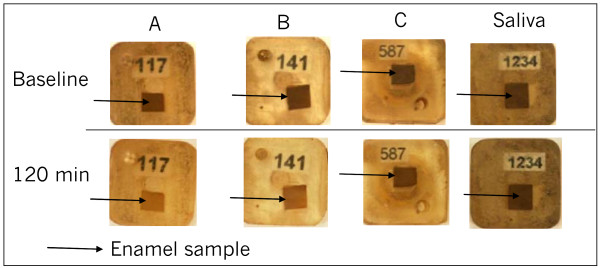
**Qualitative evaluation of the stain removal**. Stain removal by the tested product before (baseline) and after 120 min of *in vitro *mastication.

Since the human eye can visually detect a ΔE color difference of 2 or more, the amount of stain removed by chewing gums A and B produced a significant change in the enamel sample color as shown in Figure 3.

### Analysis of the quantitative evaluation

**Figure 4:**
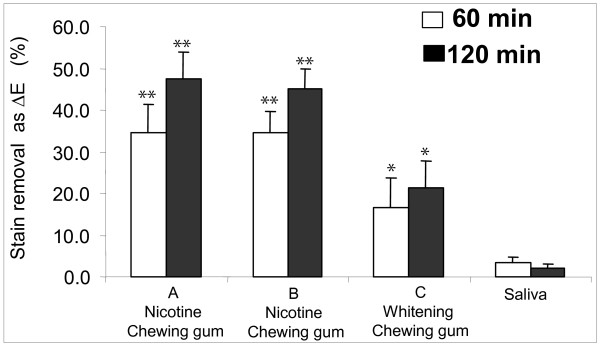
**Quantitative evaluation of the stain removal**. Percentage of total stain removed as overall change color (ΔE) by various chewing gums (A, B and C) and freshly stimulated saliva after 60 min and 120 min of *in vitro *mastication (mean ± standard deviation of 16 samples). Note: Bars with the same number of asterisks at the same time point are not statistically different. Gums A and B versus C or saliva, as well as C versus saliva, are significantly different (at p < 0.01) at 60 and 120 min.

In order to calculate the percentage of stain removed by the chewing gum, all stain remaining on the test enamel sample after the chewing phase was removed by pumicing them totally clean. An average maximum ΔE score was then calculated, which represented the total amount of removable stain on the enamel sample. Based on this score, chewing gum A removed 34.6 ± 6.9% and 47.6 ± 6.2% of the stain after 60 and 120 minutes and chewing gum B removed 34.6 ± 5.0% and 45.2 ± 4.8% of the stain after 60 and 120 minutes of mechanical chewing, respectively (Figure 4). Both were significantly more effective than chewing gum C (16.5 ± 7.4% and 21.4 ± 6.4%) and the saliva control, which removed 3.3 ± 1.4% of the stain at 60 min and 2.1 ± 1.0% at 120 min. A similar trend was observed when the percent of stain removed was expressed as change in lightness (ΔL; data not shown).

## Discussion

In this study, the reliability of results could not be influenced by the flour of pumice or the air drying of enamel specimens. The flour of pumice used for the removal of existing extrinsic stain (to determine the inherent color of the specimen), does impart a quantifiable polish to the specimen surface and it is similar to the prophylaxis paste used for dental cleanings in the dental office. Therefore, this would not cause surface scratching, which would result in scattered light, that could possibly interfere with the spectrophotometric measurement. In addition, it is relevant to note, that the specimens lighten as they dry, and that within the first few minutes, the color parameters change dramatically but stabilize after 30 minutes. Care was taken to ensure that all specimens were read as a group and allowed to dry for 30 minutes. Because all of the samples were handled in the same manner and for the same length of time, dessication of samples should not be expected to affect results. Good correlation was obtained between the overall change in the color stain (ΔE) and the lightness stain (ΔL), which influences whitening appearance of the enamel sample. Although the spectrophotometer reflectance can quantify color in an objective way, this system is not able to take spatial measurements of a specific stained area.

The medicated chewing gums A and B removed significantly greater amount of extrinsic stain than the positive control, which was still more effective than the saliva. Continued chewing from 60 to 120 min resulted in additional stain removal for all chewing gums. Stain removal evaluation of these changes was clearly evident for the medicated chewing gums A and B, but less of a difference was observed for the confectionary whitening gum C. In contrast, saliva had no detectable effect, which could have been expected due to the absence of whitening agents, such as calcium carbonate or sodium bicarbonate. This latter, known as baking soda, is soluble in saliva and classified as a mild abrasive. It improves tooth lightness by removing extrinsic tooth stain when incorporated into chewing gums [9] and intrinsic stain when added in dentifrices [10]. Calcium carbonate, on the other hand, is almost insoluble in water and forms strong abrasive particles which help to reduce plaque from teeth and improve tooth lightness by polishing the enamel surface [11-13]. Without having access to the detailed formulation of the confectionary whitening gum (C), calcium carbonate and sodium bicarbonate were identified in the 3 tested chewing gums but sodium carbonate was only found in the NRT chewing gums. Subsequently the additional presence of this molecule could contribute to the differences in whitening effect reflected by the higher stain removal with the NRT chewing gums A and B. The calcium present in saliva may react with the carbonate from sodium carbonate, resulting in formation of insoluble calcium carbonate, which has polishing properties [11-13]. Similar tooth whitening effects could be assumed for all other flavoured Nicotinell chewing gums, which contain the same amount of calcium carbonate, bicarbonate sodium and sodium carbonate as Nicotinell Mint coated and Nicotinell Mint Thrive chewing gums used in this study. The confectionary gum does not contain sodium carbonate but xylitol which is a known anticariogenic agent [14] and carbamide (urea). This latter, when used in chewing gum, demonstrated to induce a more pronounced pH recovery after an intake of sucrose [15]. Thus, xylitol and carbamide in chewing gum allow the reduction of dental plaque formation.

Dental plaque is a microbial biofilm with a diverse composition which deposits on the tooth, and is a source of dental caries and gingivitis [16,17]. In cigarette smoke, tar, arsenic, cadmium and 4000 chemicals and gases are released [18]. Depending on the length of exposure to these chemicals, it can result in extrinsic brown to dark stains on the tooth surfaces of heavy smokers, which are incorporated into the salivary pellicle [19]. Enamel pellicle is a protein film, sourcing from the continuous exposure of enamel to whole saliva, which functions to protect the enamel. Several studies have shown pellicle to drastically reduce enamel erosion in presence of acidic beverages [20]. Combination of chewing gum and abrasive ingredients might help to stimulate saliva production and mechanical removal of plaque, hence favouring renewal of the pellicle and enhancing the enamel lightness, as reported in the literature [21,22].

Medicated chewing gums containing nicotine are designed to be used as oral substitutes to cover the former smoker's nicotine needs. The smoking population has a higher prevalence of dental problems such as periodontitis and loss of teeth [23]. Thus, the present results provide evidence that the NRT tested chewing gums can contribute to a better overall oral hygiene by removing the stained pellicle on the enamel surface, hence increasing the tooth whitening appearance. This effect might be stressed by healthcare professionals when advising their patients to stop smoking, since esthetic considerations are often cited by patients as one among many reasons to comply with professional advice [24].

Considering all the above points, the *in vitro *mastication system is a suitable method for assessing the mechanical removal of extrinsic stain by abrasive or polishing agents such as sodium bicarbonate and carbonate calcium respectively. However this procedure might not allow to underscore the effect of agent like xylitol or carbamide on reduced dental plaque formation, which contribute also to tooth whitening appearance [11-13].

## Conclusion

These results show the *in vitro *mastication system to be a valid and reliable method to evaluate the removal of extrinsic stain on enamel. NRT chewing gums of the Nicotinell brand were significantly more effective than a confectionary chewing gum claiming to have a whitening effect, and saliva alone. This visible tooth whitening action could strengthen the smokers' motivation to use adequate NRT support when planning to quit smoking.

## Competing interests

GS and NHN are employees of Novartis Consumer Health, which produces and distributes the Nicotine replacement therapy products. This study was funded by Novartis Consumer Health.

The authors declare that they have no competing interests.

## Authors' contributions

MM was the supervisor of the project and carried out the investigations and evaluated results.  NH–N supported the experiments and contributed to the evaluation of results, and writing of the manuscript. GS contributed to the draft manuscript and revised it critically for relevant intellectual content. All authors have read and approved the final version of the manuscript.

## Pre-publication history

The pre-publication history for this paper can be accessed here:


